# Supplementary material for: Organic Amendments and Trichoderma Change the Rhizosphere Microbiome and Improve Cucumber Yield and Fusarium Suppression
Source: Plants (Basel). 2025 Dec 1;14(23):3660. doi: 10.3390/plants14233660 (PMC12694271; doi:10.3390/plants14233660)
Supplement: Supplementary file 1 [file plants-14-03660-s001.zip › plants-3994621-supplementary.pdf]

Supplementary

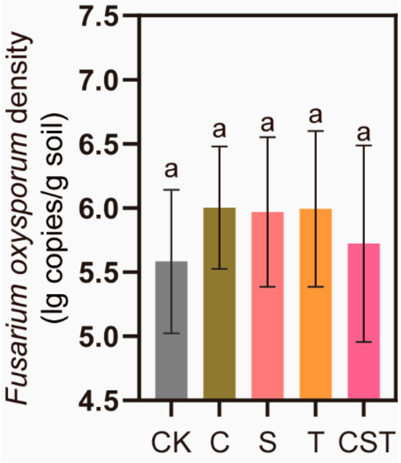

Figure S1. The pathogen density in the field experiment before applying different treatments.

CK denotes control treatment; C denotes chitin addition treatment; S denotes straw addition treatment; T denotes the *Trichoderma* strain (*T. guizhouense* NJAU4742) addition treatment; CST denotes treatment with a mixture of straw, chitin and the *Trichoderma* strain (T. *guizhouense* NJAU4742). n = 9 and data are presented as mean ± SD. Statistical significance was calculated by Duncan's test. Different letters represent a significant difference at $P < 0.05$ according to Duncan's test.

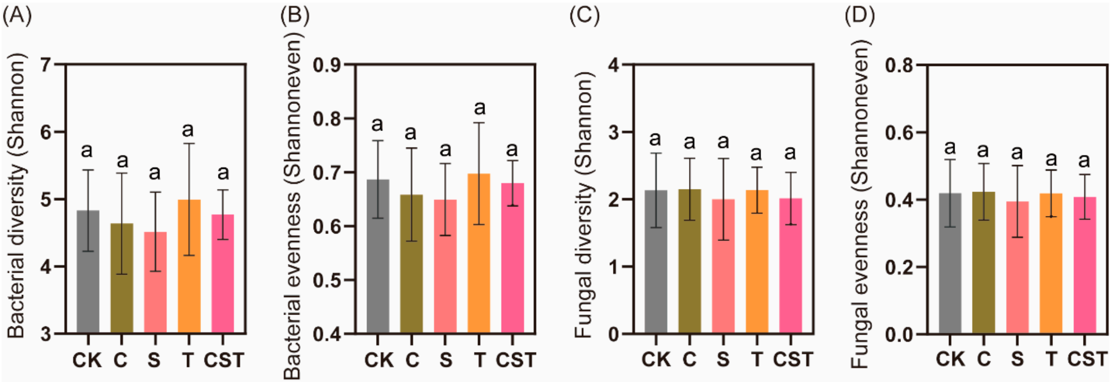

Figure S2. Effects of the addition of different materials on the rhizosphere microbial diversity and evenness.

CK denotes control treatment; C denotes chitin addition treatment; S denotes straw addition treatment; T denotes the *Trichoderma* strain (*T. guizhouense* NJAU4742) addition treatment;

CST denotes treatment with a mixture of straw, chitin and the *Trichoderma* strain (T. *guizhouense* NJAU4742). n = 9 and data are presented as mean ± SD. Statistical significance was calculated by Duncan's test. Different letters represent a significant difference at $P < 0.05$ according to Duncan's test.

Table S1 Relationship between microbial α-diversity and cucumber yield

		Yield
Bacterial diversity（Shannon）	R^2	-0.145
	P	0.342
Bacterial evenness（Shannoneven）	R^2	-0.154
	P	0.311
Fungal diversity（Shannon）	R^2	-0.120
	P	0.431
Fungal evenness（Shannoneven）	R^2	-0.081
	P	0.596

Table S2 Relationship between soil microbal α-diversity and *Fusarium oxysporum* density

		Fusarium oxysporum density
Bacterial diversity（Shannon）	R^2	0.103
	P	0.503
Bacterial Evenness（Shannoneven）	R^2	0.125
	P	0.413

Fungal diversity（Shannon）	R^2	0.064
	P	0.677
Fungal Evenness（Shannoneven）	R^2	0.014
	P	0.926

Table S3 Treatment effects on bacterial community composition based on ANOSIM.

	Bacterial community composition
CK vs C	ANOSIM=0.834; *P*=0.003
CK vs S	ANOSIM=0.801; *P*=0.004
CK vs T	ANOSIM=0.811; *P*=0.004
CK vs CST	ANOSIM=0.953; *P*<0.001
C vs S	ANOSIM=0.223; *P*=0.530
C vs T	ANOSIM=0.137; *P*=0.641
C vs CST	ANOSIM=0.577; *P*=0.023
S vs T	ANOSIM=0.113; *P*=0.663
S vs CST	ANOSIM=0.571; *P*=0.021
T vs CST	ANOSIM=0.698; *P*=0.015

CK denotes control treatment; C denotes chitin addition treatment; S denotes straw addition treatment; T denotes the *Trichoderma* strain (*T. guizhouense* NJAU4742) addition treatment; CST denotes treatment with a mixture of straw, chitin and the *Trichoderma* strain (T. *guizhouense* NJAU4742).

Table S4 Treatment effects on fungal community composition based on ANOSIM.

	Fungal community composition
CK vs C	ANOSIM=0.854; *P*=0.004
CK vs S	ANOSIM=0.879; *P*=0.002
CK vs T	ANOSIM=0.873; *P*=0.002

CK vs CST	ANOSIM=0.946; *P*<0.001
C vs S	ANOSIM=0.343; *P*=0.427
C vs T	ANOSIM=0.241; *P*=0.534
C vs CST	ANOSIM=0.638; *P*=0.012
S vs T	ANOSIM=0.180; *P*=0.658
S vs CST	ANOSIM=0.588; *P*=0.036
T vs CST	ANOSIM=0.670; *P*=0.022

CK denotes control treatment; C denotes chitin addition treatment; S denotes straw addition treatment; T denotes the *Trichoderma* strain (*T. guizhouense* NJAU4742) addition treatment; CST denotes treatment with a mixture of straw, chitin and the *Trichoderma* strain (T. *guizhouense* NJAU4742).

Table S5 Relationship between soil microbial community composition and cucumber yield

		Yield
Bacterial community composition（PCoA1）	R^2	0.785
	P	<0.001
Fungal community composition（PCoA1）	R^2	0.722
	P	<0.001

Table S6 Relationship between soil microbial community composition and *Fusarium oxysporum* density

		Fusarium oxysporum density
Bacterial community composition（PCoA1）	R^2	-0.752

	P	<0.001
Fungal community composition（PCoA1）	R^2	-0.623
	P	<0.001

Table S7 Bacterial OTUs that have positive correlations with cucumber yield

		Yield
OTU6610	R^2	0.523
(*Niabella*)	*P*	<0.05
OTU12	R^2	0.491
(*Streptacidiphilus*)	*P*	<0.05
OTU4748	R^2	0.475
(*Parasegetibacter*)	*P*	<0.05
OTU2398	R^2	0.470
(*Rathayibacter*)	*P*	<0.05
OTU4454	R^2	0.427
(*Amnibacterium*)	*P*	<0.05
OTU412	R^2	0.427
(*Tepidibacillus*)	*P*	<0.05
OTU133	R^2	0.421
(*Pedobacter*)	*P*	<0.05
OTU642	R^2	0.415
(*Nakamurella*)	*P*	<0.05
OTU10431	R^2	0.400
(*Lysinibacillus*)	*P*	<0.05
OTU336	R^2	0.398
(*Phenylobacterium*)	*P*	<0.05

OTU53	R^2	0.388
(*Sphaerobacter*)	*P*	<0.05
OTU5166	R^2	0.380
(*Tumebacillus*)	*P*	<0.05
OTU1025	R^2	0.376
(*Streptomyces*)	*P*	<0.05
OTU653	R^2	0.370
(*Propionicicella*)	*P*	<0.05
OTU312	R^2	0.369
(*Nocardia*)	*P*	<0.05
OTU552	R^2	0.366
(*Nakamurella*)	*P*	<0.05
OTU615	R^2	0.360
(*Nakamurella*)	*P*	<0.05
OTU8096	R^2	0.350
(*Ornithinibacillus*)	*P*	<0.05
OTU10763	R^2	0.345
(*Fulvimonas*)	*P*	<0.05
OTU3672	R^2	0.343
(*Streptomyces*)	*P*	<0.05
OTU1978	R^2	0.335
(*Parasegetibacter*)	*P*	<0.05
OTU189	R^2	0.331
(*Pallidibacillus*)	*P*	<0.05
OTU347	R^2	0.328
(*Cohnella*)	*P*	<0.05

OTU433	R^2	0.326
(*Streptomyces*)	*P*	<0.05
OTU42	R^2	0.323
(*Candidimonas*)	*P*	<0.05
OTU3552	R^2	0.321
(*Gp3*)	*P*	<0.05
OTU5452	R^2	0.317
(*Sphingomonas*)	*P*	<0.05
OTU649	R^2	0.315
(*Sphaerobacter*)	*P*	<0.05
OTU1083	R^2	0.311
(*Bacillus*)	*P*	<0.05
OTU13549	R^2	0.308
(*Bacillus*)	*P*	<0.05
OTU452	R^2	0.305
(*Camellibacillus*)	*P*	<0.05
OTU94	R^2	0.305
(*Bacillus*)	*P*	<0.05
OTU110	R^2	0.305
(*Oryzihumus*)	*P*	<0.05
OTU491	R^2	0.303
(*Natronotalea*)	*P*	<0.05
OTU445	R^2	0.301
(*Rhodovibrio*)	*P*	<0.05
OTU1819	R^2	0.300
(*Bacillus*)	*P*	<0.05

OTU855	R^2	0.299
(*Flavisolibacter*)	*P*	<0.05

Table S8 Bacterial OTUs that have negative correlations with *Fusarium oxysporum* density

		Fusarium oxysporum density
OTU13549	R^2	-0.436
(*Bacillus*)	*P*	<0.05
OTU449	R^2	-0.428
(*Altererythrobacter*)	*P*	<0.05
OTU12	R^2	-0.417
(*Streptacidiphilus*)	*P*	<0.05
OTU4922	R^2	-0.409
(*Halobacillus*)	*P*	<0.05
OTU433	R^2	-0.383
(*Falsiroseomonas*)	*P*	<0.05
OTU200	R^2	-0.368
(*Rhodanobacter*)	*P*	<0.05
OTU496	R^2	-0.345
(*Bacillus*)	*P*	<0.05
OTU6610	R^2	-0.338
(*Niabella*)	*P*	<0.05
OTU1025	R^2	-0.336
(*Streptomyces*)	*P*	<0.05
OTU6017	R^2	-0.331
(*Acidovorax*)	*P*	<0.05
OTU4454	R^2	-0.330

(*Amnibacterium*)	*P*	<0.05
OTU759	R^2	-0.314
(*Cerasibacillus*)	*P*	<0.05
OTU29	R^2	-0.311
(*Dyella*)	*P*	<0.05
OTU855	R^2	-0.311
(*Flavisolibacter*)	*P*	<0.05
OTU216	R^2	-0.310
(*Bacillus*)	*P*	<0.05
OTU2398	R^2	-0.300
(*Rathayibacter*)	*P*	<0.05
OTU506	R^2	-0.299
(*Actinocatenispora*)	*P*	<0.05
OTU312	R^2	-0.297
(*Nocardia*)	*P*	<0.05
OTU231	R^2	-0.296
(*Pseudalkalibacillus*)	*P*	<0.05
OTU747	R^2	-0.295
(*Symbioplanes*)	*P*	<0.05
OTU251	R^2	-0.295
(*Bacillus*)	*P*	<0.05

Table S9 Fungal OTUs that have positive correlation with cucumber yield

		Yield
OTU313	R^2	0.503
(*Trichoderma*)	*P*	<0.05

OTU8	R^2	0.483
(*Mortierella*)	*P*	<0.05
OTU1067	R^2	0.441
(*Mortierella*)	*P*	<0.05
OTU46	R^2	0.441
(*Trichoderma*)	*P*	<0.05
OTU17	R^2	0.368
(*Thoreauomyces*)	*P*	<0.05
OTU105	R^2	0.309
(*Penicillium*)	*P*	<0.05

Table S10 Fungal OTUs that have negative correlation with *Fusarium oxysporum* density

		Fusarium oxysporum density
OTU8	R^2	-0.557
(*Mortierella*)	*P*	<0.05
OTU1067	R^2	-0.504
(*Mortierella*)	*P*	<0.05
OTU313	R^2	-0.500
(*Trichoderma*)	*P*	<0.05
OTU46	R^2	-0.490
(*Trichoderma*)	*P*	<0.05
OTU25	R^2	-0.323
(*Corynespora*)	*P*	<0.05
OTU60	R^2	-0.315
(*Kochiomyces*)	*P*	<0.05
OTU17	R^2	-0.314

（*Thoreauomyces*）	*P*	<0.05
OTU31	R^2	-0.311
（*Rhizophlyctis*）	*P*	<0.05
